# Microwave-Assisted vs. Conventional Extraction of *Moringa oleifera* Seed Oil: Process Optimization and Efficiency Comparison

**DOI:** 10.3390/foods13193141

**Published:** 2024-10-01

**Authors:** Danivia Endi Santana Souza, Jéssica Jessi Carvalho de Melo, Fernanda Franca dos Santos, Ana Luíza dos Santos Vasconcelos, Adriana dos Santos de Jesus, Lisiane dos Santos Freitas, Ranyere Lucena de Souza, Cleide Mara Faria Soares

**Affiliations:** 1 Postgraduate Program Process Engineering, Tiradentes University (UNIT), Campus Farolandia, Aracaju 49032-490, SE, Brazil; danivia.endi@souunit.com.br (D.E.S.S.); jessica.jcarvalho@hotmail.com (J.J.C.d.M.); fernanda.fdsantos@souunit.com.br (F.F.d.S.); ana.luiza03@souunit.com.br (A.L.d.S.V.); adriana.jesus81@souunit.com.br (A.d.S.d.J.); ranyerels@hotmail.com (R.L.d.S.); 2 Department of Chemistry, Federal University of Sergipe (UFS), São Cristóvão 49100-000, SE, Brazil; lisiane@academico.ufs.br; 3 Institute of Technology and Research (ITP), Aracaju 49032-490, SE, Brazil

**Keywords:** *Moringa oleifera* Lam. seeds, microwave-assisted, oil yield, response surface methodology, oleic acid

## Abstract

This study aims to evaluate the effectiveness of microwave-assisted and conventional extraction using ethanol, hexane, and petroleum ether as solvents, and to optimize the process for extracting oil from *Moringa oleifera* Lam. seeds, with a focus on improving food-grade oil production. Response surface methodology (RSM) was applied to enhance the extraction process of the oil. Central composite rotational design (CCRD) was used to analyze the impact of solid–liquid ratio (x_1_), power (x_2_), and temperature (x_3_) on oil yield. The optimization identified the optimal conditions as a solid/liquid ratio of 1:38, power of 175 W, and temperature of 50 °C, achieving a 42% oil yield. Notably, the microwave-assisted extraction reduced the processing time from 8 h (using conventional Soxhlet extraction) to just 1 h. Conventional extraction with hexane and petroleum ether was also performed for comparison, resulting in similar oil content and fatty acid profiles, predominantly, oleic acid. FTIR analysis confirmed that the microwave-extracted oil contained fatty acids and had similar characteristics to the conventionally extracted oil. Thus, the use of ethanol as a green solvent in the microwave has shown significant improvement in terms of time and energy savings compared to the Soxhlet method with toxic solvents. This study concludes that microwave-assisted extraction with ethanol provides a more energy efficient, environmentally friendly, and time-saving alternative for food-grade oil production, aligning with advancements in food engineering and production.

## 1. Introduction

The comparison of microwave-assisted versus conventional extraction techniques in oil processing can lead to improvements in product quality and process efficiency. The rationale is that microwave technology shows potential for intensifying the extraction of natural products. According to the literature, the advantage of the industrial application of microwave technology over conventional processing is process automation and the use of smaller equipment with faster start-up and shutdown times. Thus, microwave-assisted extraction is an alternative to improve energy efficiency in the process for obtaining the bioproducts, such as food, biomedical and pharmaceutical products [1,2]. Advances in this area highlight the need for research into innovative biomass conversion technologies that can enable more sustainable processes [3]. 

Natural and renewable resources include oilseeds, which are becoming increasingly important as a disaster risk-reduction solution in agriculture due to their tolerance to drought and environmental stress. Oilseeds are a food source due to their high nutritional value, and they also have a variety of non-food applications [4]. *Moringa oleifera* Lam. seed oil, for example, is an alternative highlighted for potential pharmaceutical and industrial uses such as cosmetics, medicine, biofuel, and biolubricants [5,6]. *Moringa oleifera* Lam is an evergreen, fast-growing, drought-resistant tree with multifunctional properties due to its nutritional richness. Compounds of interest include fatty acids, proteins, fibers, minerals, flavonoids, phenolic acids, glycosides, phytosterols, alkaloids, glucosinolates, thiocarbamates, and tocopherols [7]. 

The literature reports that the oil yield extraction from these seeds is 39% using Soxhlet with hexane as solvent [6]. The oil obtained from *Moringa oleifera* seeds is called Ben oil, which is a highly qualified substitute for olive oil composed of fatty acids, mainly oleic acid (76%) [6,8,9]. Oils with a high oleic acid content are becoming increasingly significant, particularly as a substitute for polyunsaturated vegetable oils. Oleic acid is an unsaturated acid that confers good thermal and oxidative stability to the oil [10].

In this context, there is an opportunity to explore and develop a new source of high-quality oil from *Moringa oleifera* seeds using innovative and more sustainable extraction technologies [11]. The most efficient conventional method for extracting oil is solid–liquid extraction by Soxhlet, which requires use of toxic solvents. Therefore, the objective is to enhance existing methodologies in a manner that is both effective and environmentally responsible. In this regard, a variety of techniques for solid–liquid extraction may be employed, including microwave-assisted extraction (MAE), ultrasound-assisted extraction (UAE), pressurized solvent extraction (PSE), and two-part aqueous extraction (ATPE) [12]. 

Among these methods, MAE stands out for its ability to accelerate the processing of the biomass and enable automation and control of variables process. Although the oilseeds are made up of nonpolar macromolecules generally insoluble in organic solvents, MAE emerges as an alternative due to the thermal effects generated by the energy in the reactor, initiating the dissolution of the solvent and solute. The molecules vibrate because of electromagnetic waves, creating internal heat and promoting mass transfer from the inside to the outside [13]. Consequently, it may be possible to reduce extraction times and temperature, conserving the oil yield and composition of fatty acids. Moreover, this reduction leads to greater oxidative stability, preserving the concentration of polyunsaturated fatty acids [14].

The process results in the dissipation of energy in dipole polarization and ionic conduction molecules, which favors the use of polar solvents such as ethanol [15]. Among the solvents used in extraction, ethanol is a recommended low-cost green alternative when compared to hexane and petroleum ether, considered environmentally hazardous [16]. Microwave-assisted ethanol oil extraction will enable a more sustainable and time-saving alternative solid–liquid extraction process without losing oil extraction efficiency, while still preserving the composition of the molecules of interest. This results in a reduction in the costs associated with energy and solvents, which, in turn, leads to a decrease in the overall cost of the process [10,15].

The effects of the variables are of paramount importance for the optimization of the process in terms of oil yield. The main variables in microwave-assisted extraction are the solid–liquid ratio, microwave power (W), and temperature (°C) [17]. To enhance the efficacy of data analysis, these variables can be employed in response surface methodology (RSM) based on a central composite rotating design (CCRD). The CCRD can be employed to investigate the interaction between the effects of varying the independent variables in the dependent variable, namely, in this investigation, oil yield. The optimal region of the process can be identified through the interpretation of the statistical data obtained from the RSM, based on the selected variables [18,19].

Moreover, the physicochemical analysis of the oil via Fourier transform infrared spectroscopy (FTIR) enables the identification of functional groups based on the alterations observed in the infrared spectrum [20]. Subsequently, gas chromatography (GC) can be employed to quantify the presence of fatty acids [21].

Therefore, the objective of this study is to optimize the oil yield (%) of microwave-assisted extraction using ethanol to produce *Moringa oleifera* oil with a high oleic acid content. Comparing the oil obtained by a conventional method using toxic solvents is essential to validate the data.

## 2. Materials and Methods

### 2.1. Materials and Reagents

The *Moringa oleifera* Lam. seeds were collected from the EMBRAPA (Brazilian Agricultural Research Corporation), located in Sergipe, Brazil (latitude 10°57′14″ S and longitude 37°03′79″). The solvents used ethanol and n-hexane (both 95% P.A. ACS), were purchased from NEON, São Paulo, Brazil. Petroleum ether (30–60 °C, P.A. ACS) was purchased from ACS Scientific, São Paulo, Brazil. 

### 2.2. Preparation of Moringa oleifera Seeds

*Moringa oleifera* Lam. mature seeds were obtained from the Farolândia Campus of the Tiradentes University (UNIT) in Aracaju, Sergipe, Brazil. The collection was conducted during the summer months, at which point the plants were observed to have reached full maturity. Subsequently, the seeds were subjected to immediate processing to ensure the highest quality of the final product. The seeds were subjected to a 5 h drying process at 80 °C. The moisture content was determined both before and after the drying process using a Shimadzu Halogen Infrared moisture balance (model MOC63u). The final moisture content was 3%; this content facilitated the grinding process, which was conducted using a sieve with a size of over 60 mesh to produce a fine and uniform powder. The seed triturates were stored at room temperature with humidity levels controlled [8].

### 2.3. Microwave-Assisted Oil Extraction from Moringa oleifera Seeds

*Moringa oleifera* seeds were irradiated using ethanol as the solvent in a microwave reactor (Model Discover SP, CEM Corporation, Matthews, NC, USA), with a fixed time of 60 min set in the Synergy Application software. The mass–solvent ratio, power, and temperature (°C) varied under agitation. The oil extraction protocol was based on the methodologies of Nebolisa et al. (2023) [17], Xingnan et al. (2022) [18], and Zhong et al. (2018) [22] with some modifications. After the extraction process, the samples were evaporated in a vacuum rotary evaporator (Tecnal, TE-211) at 60 °C to remove the solvent. The oil yield (%) will be calculated according to Fu et al. (2021) [23] using Equation (1) as described below:

(1) $$Oil\;Yield\left(\%\right)=\frac{mass\;of\;extracted\;oil\;(g)}{initial\;mass\;of\;seeds\;(g)}\times 100\%$$

### 2.4. Conventional Oil Extraction from Moringa oleifera Seeds

Soxhlet extraction was conducted for comparative purposes with conditions like those optimized for microwave extraction. Ethanol, hexane, and petroleum ether were used as solvents, at the same optimized solid–liquid ratio. The extraction time was 8 h and the temperature was according to the boiling point of each solvent. This methodology was followed, utilizing the data from Barbosa et al. (2021) [8], for this specific equipment.

### 2.5. Design of Experiments

The experimental design was conducted with the aim of optimizing the oil extraction process from *Moringa oleifera* Lam seeds. Independent variables were selected: solid–liquid ratio (*m*/*v*) (x_1_), power (W) (x_2_), and temperature (°C) (x_3_). The study evaluated their effect on the response variable (Y), expressed as oil yield (%). 

Initially, the effects of the independent variables on the oil yield were evaluated using a Central Composite Rotational Design (CCRD) 2^3^ with five levels, coded according to Table 1. The variables selected are those commonly studied in microwave processes, and the values were determined based on research by Chen et al. (2017) [13] and Xingnan et al. (2022) [18], taking into consideration the maximum power and temperature limits set by the equipment.

Thus, to optimize the oil yield, we used the CCRD 2^2^ and analyzed the results shown in Table 1. The investigation evaluated superior solid/liquid ratio (*m*/*v*) values at power settings like those in our previous design.

### 2.6. Statistical Analysis and Optimization

Statistical analysis of the data was conducted using the online software Protimiza Experimental Design (http://experimental-design.protimiza.com.br/; accessed on 1 July 2024). The second-order polynomial model was used to fit the responses (Y), with the regression coefficients of the linear, quadratic, and interaction variables (x_1_, x_2,_ and x_3_) as per Equation (2):

(2) $$Y=\beta_{0}+\beta_{1}x_{1}+\beta_{2}x_{2}+\beta_{3}x_{3}+\beta_{11}x_{1}^{2}+\beta_{22}x_{2}^{2}+\beta_{33}x_{3}^{2}+\beta_{12}x_{1}x_{2}+\beta_{13}x_{1}x_{3}+\beta_{23}x_{2}x_{3}$$

where Y is the measured response; *β*_0_ is the intercept (constant); and *β*_1_ to *β*_3,3_ are the coefficients associate with linear, quadratic and interaction effects, respectively, of variables x_1_, x_2,_ and x_3_, respectively. The models study the effect of each independent variable and all interactions between them on a particular response. 

The significance model was evaluated using analysis of variance (ANOVA). The adequacy of the polynomial model equation was expressed by the coefficient of determination (R^2^), and its statistical significance was confirmed by the F-test. Additionally, the significance of the regression coefficients was confirmed by the F-test. A *p*-value of 0.05 was set as the level of significance. The experiments were conducted in triplicate.

Response surface methodology (RSM) was applied to investigate the effect of the studied variables on the response variable (Y) and to define the optimal range of the extraction process. Finally, the experiment was conducted in triplicate under the conditions of the calculated optimum point to validate the mathematical model obtained.

### 2.7. Comparative Analysis of Oil Extracted from Moringa oleifera Seeds

The comparative analysis evaluated the efficiency of oil extraction from *Moringa oleifera* Lam. seeds using a microwave-assisted method with ethanol as the green solvent, with the conventional method, by Soxhlet. In addition, the most-used solvents, hexane and petroleum ether, were also evaluated in combination with the methods. The extractions were conducted based on the optimal conditions previously established for microwave-assisted extraction with ethanol.

The combinations of extraction methods and solvents were Microwave (MW) with ethanol (MW-Ethanol), hexane (MW-Hexane), and petroleum ether (MW-P.Ether); and Soxhlet (SO) with ethanol (SO-Ethanol), hexane (SO-Hexane), and petroleum ether (SO-P.Ether). Thus, the comparison was made based on the data of quantification of fatty acids (%), oil yield (%) (Equation (1)), and physicochemical analysis, as described in the topics below.

#### 2.7.1. Gas Chromatography Analysis

The samples of *Moringa oleifera* oil were quantified by gas chromatography using a GC-FID 2010 apparatus (Shimadzu, Japan) with a SUPELCOWAX10 column (30 m × 0.25 mm × 0.25 µm), by the methodology Almeida et al. (2020) [21]. The oven temperature was initially set at 70 °C for 3 min, then increased to 210 °C at a rate of 24 °C/min, followed by an additional increase to 230 °C at a rate of 5 °C/min, where it was maintained for 13 min. Nitrogen was employed as the carrier gas at a rate of 1.7 mL/min. The injector was operated in split mode (1:20) at a temperature of 230 °C, with an injection volume of 1 μL. 

The FAME Mix, C4-C24 (18919-1AMP, Sigma-Aldrich Poznan, Poland) was used as a standard because it contains 37 fatty acid methyl esters. To determine the fatty acid content in the oil samples, the methyl esters present were identified by comparison with the retention times of FAME. In the sample with FAME, a correlation factor was calculated for all methyl esters of fatty acids present in *Moringa oleifera* oil. The internal standard used was methyl heptadecanoate (C17:0), based on the mass of FAME and the corresponding peak areas for each fatty acid found.

The samples were prepared with a total of 300 μL of the oil derivatization product, 250 μL of the internal standard (C17:0, at a concentration of 1000 mg/L), and 450 μL of n-hexane (grade for GC), transferred to a vial with a final volume of 1000 μL. An aliquot of 1 μL of the sample was injected into the GC-FID system. Injections were performed in duplicate for all samples. 

#### 2.7.2. Fourier Transform Infrared Spectroscopy Analysis 

The spectra of *Moringa oleifera* Lam. seed oil was obtained via Fourier transform infrared spectroscopy (FTIR) on a Cary 630 FTIR spectrometer (Agilent Technologies, Waldbronn, Germany). The analyses were conducted to compare the oil extracted by the microwave-assisted method with ethanol to the more commonly used method, the Soxhlet method with hexane. The spectrum was obtained at 25 °C within a spectral range of 4000–650 cm^−1^ with a spectral resolution of 4 cm^−1^ [20]. The FTIR bands were assigned to a specific functional group by the findings of previous studies.

## 3. Results

### 3.1. Effect of Variables of Microwave-Assisted on Moringa oleifera Oil Yield

The experimental design initially investigated the effect on microwave-assisted oil yield by varying the solid–liquid ratio (*m*/*v*) (x_1_), power (W) (x_2_), and temperature (°C) (x_3_). The obtained response results were fitted into Equation (2), constituting a second-order model. Table 2 shows 18 tests, among which was a 2^3^ full factorial design, including six axial points and four repetitions at the central point, as well as the dependent variable Y, as a response obtained by the percentage oil yield. 

The results from the full factorial design (1–8) demonstrate a significant effect on the response with increasing solid/liquid ratio, and, more subtly, with increasing power. Nevertheless, elevated temperature had no effect on the oil yield. The axial point data confirms these data (9–14), while the central points can evaluate the experimental error. The findings presented in Table 2 demonstrate that the maximum molar ratios (1:20 and 1:24) can achieve the highest oil extraction efficiencies for this experimental design. The highest oil yield values were found at the highest power levels (250 and 300 W), but significant values were also found at lower power levels (100 and 175 W). 

Based on these results, it can be concluded that an increase in the magnitude of significant variables leads to an increase in oil yield efficiency. Therefore, the higher x_1_ and x_2_, the higher the percentage oil yield seems to be. However, the varying power levels produced relevant results for oil yield, which requires further study. Another significant aspect to consider in terms of the solid–liquid ratio effect on response is the lower yield values, as evidenced by (Runs 1, 3, 5, 7, and 9), which were provided at low solid–liquid ratios (1:4 and 1:8). 

Based on these results, it can be concluded that an increase in the magnitude of significant variables leads to an increase in oil extraction efficiency. Therefore, the higher x_1_ and x_2_, the higher the percentage oil yield seems to be. However, the varying power levels produced relevant results for oil extraction, which requires further study. Another significant aspect to consider in terms of the solid–liquid ratio effect on response is the lower yield values, as evidenced by (Runs 1, 3, 5, 7, and 9), which were provided at low solid-liquid ratios (1:4 and 1:8). 

**Figure 1:**
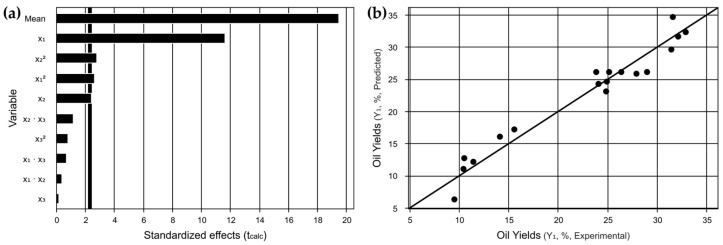
CCRD 2^3^ graphs. (**a**) Pareto graph of t-values showing the effects of independent variables and their interactions on the response variable. (**b**) Comparison of predicted values and actual values obtained experimentally.

These data are confirmed in the Pareto plot shown in Figure 1a, where the linear and quadratic factors of x_1_ and x_2_ are shown as significant and x_3_ is shown as not significant because it is not within the established significance level (<0.05). Additionally, the Figure 1b correlation graph depicts an agreement between the predicted and actual experimental results. Another relevant point is the coefficient of determination obtained, which has a confidence level with R^2^ equal to 95.09%.

**Figure 2:**
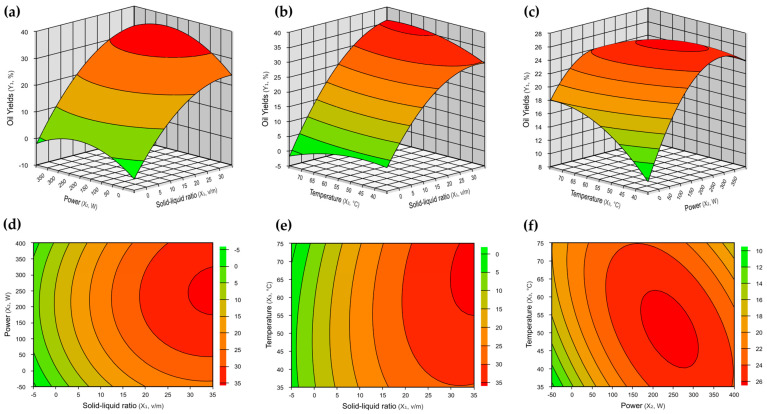
Response surface graphs (**a**–**c**) and contour curves (**d**–**f**) for oil yield. (**a**,**d**) Solid–liquid ratio and power; (**b**,**e**) solid–liquid ratio and temperature; (**c**,**f**) temperature and power.

These observations are more clearly seen in Figure 2a,b,d,e, which show that the optimum range with respect to the variable x_1_ tends to be found above the range tested. The closer to the optimal region, the more the ellipse highlighted in the red region is formed. This demonstrates the need for studies with solid–liquid ratios greater than 1:24. Furthermore, from Figure 2a,c,d,f, the optimum power region can be found above 175 W depending on the solid/liquid ratio used. The Figure 2c,f shows that the optimum temperature region can be found in the whole evaluated range, especially in the ellipse in contour curves (Figure 2f). The response surface methodology confirmed the experimental data and predicted a new study region to find the optimum region for extracting oil.

### 3.2. Optimization of Microwave-Assisted Oil Yield

In this stage of the study, we obtained the optimal region for the microwave-assisted extraction process by investigating the effect of the solid–liquid ratio (*m*/*v*) (x_1_) and the power (W) (x_2_), which were the variables selected in the previous topic due to the need to deepen the studies and find the optimal oil yield region. The dependent variable Y is represented by the percentage oil yield. Table 3 displays the CCRD matrix comprising 11 tests: the full factorial design 2^2^, four axial points, and three repetitions at the central point. The response results were subsequently adjusted to the second-order model derived from Equation (2). The polynomial regression mathematical model that defined the experimental design to find the optimized region was given by Equation (3):

(3) $$Y={38.53+9.15x}_{1}-5.52x_{1}^{2}+1.05x_{2}-1.34x_{2}^{2}$$

From the results obtained for Y, the optimal oil yield conditions were determined. In the context of a regression model, a coefficient of the linear factors (x_1_ and x_2_) with a positive sign indicates that there is a direct relationship between the independent variable and the dependent variable. Conversely, the negative sign in the coefficients of the quadratic factors ($x_{1}^{2}\;and\;x_{2}^{2})$ indicates the direction of curvature of the response surface. The data delineate the optimal region and facilitate comprehension of the influence of the independent variables on the response variable, thereby enabling process optimization. The absence of interaction variables (x_1_.x_2_) shows that one variable did not influence the effect of another variable. 

Thus, the solid–liquid ratio showed a greater impact on oil yield than power in trials 1–4 of the factorial plan, while this trend was confirmed in trials 5–8 along the axial points. The results in Table 3 are in accordance with the previous planning data presented in Section 3.1. The highest oil yield achieved was 42% in run 6, when utilizing a solid–liquid ratio of 1:38 and a power input of 175 W. Another relevant result, 39.9%, is found in run 4, when utilizing a solid–liquid ratio of 1:34 and a power input of 250 W. These results show that it may be possible to use a lower power value at a higher ratio or to use a lower ratio at a higher power and obtain a similar oil yield result, which may indicate that there is an optimum range to be found from the effect of these variables on the response. The oil yield was similar to the 39% obtained in the Soxhlet extraction system determined by Wilsthire et al. (2022) [6].

Equation (3) was used to determine the optimum point of the derivative. The calculations showed that the optimum condition can be found at a solid–liquid ratio of 1:34 and a power of 175 W, while maintaining the temperature. The experiment in this condition was then conducted, confirming the data, with oil yield results of 42%, similar to those found in the best condition of the design of experiments. The optimal solid–liquid ratio can be identified within the 1:34 or 1:38 range, which has been validated through data analysis and has demonstrated a reduction in solvent costs. About CCRD 2^3^, it was demonstrated that the utilization of elevated powers is not a prerequisite for the optimal oil yield in microwave-assisted extraction.

**Figure 3:**
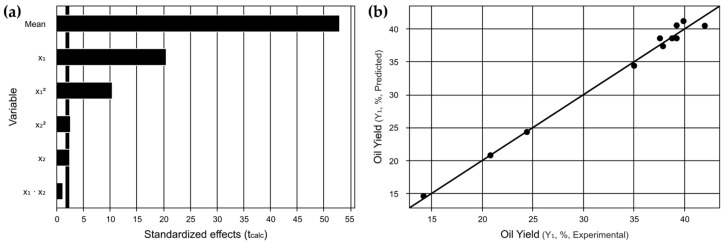
CCRD 2^2^ Graphs: (**a**) Pareto graph of t-values indicating the influences of independent variables and their interactions on the response variable. (**b**) Comparison between predicted values and actual values obtained experimentally.

Based on Figure 3a, the Pareto chart validates the significance of the linear and quadratic factors of x_1_ and x_2_, which fall within the predetermined significance level (<0.1). The linear x_1_ and quadratic $x_{1}^{2}$ factors corresponding to the solid–liquid ratio holds the most significance and demonstrate the importance of solid–liquid ratio in determining the optimal region for microwave-assisted oil extraction from *Moringa oleifera* Lam seeds. One possible explanation for this is related to mass transfer, as the greater amount of solvent facilitates this process. Meanwhile, the graph in Figure 3b illustrates a notable correlation between the predicted and actual experimental results. Additionally, the achieved coefficient of determination displays a high level of confidence with an R^2^ value of 99.08%.

In order to determine the significance and robustness of the process in the protocol used, the model was evaluated using an analysis of variance (ANOVA) and the results are shown in Table 4. The regression data showed a calculated F (107.5) which was higher than the tabulated F value (5.05). Therefore, the variation in x_1_ and x_2_ has significant effect on the response, proving that the ANOVA meets the criteria. Moreover, in relation to the lack of fit, the calculated F value of 3.1 was lower than the tabulated F value of 19.16, confirming that the results do not significantly differ from the experimental data. Therefore, based on the ANOVA analysis, the data confirm the statistical significance of the model in relation to the average response.

**Figure 4:**
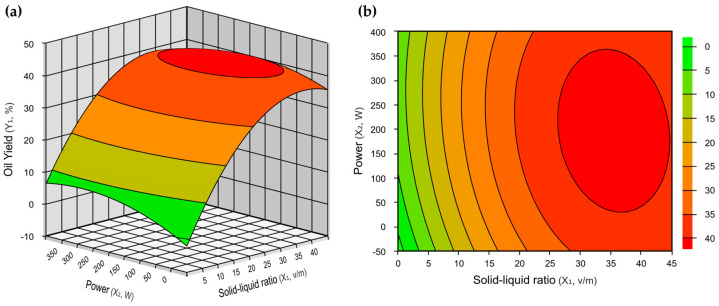
Response surface graphs (**a**) and contour curves (**b**), in relation to the effect of solid–liquid ratio (x_1_) and power (x_2_) on oil yield. Optimal microwave extraction conditions for *Moringa oleifera* oil are highlighted in red ellipse.

Figure 4 illustrates the optimum region in relation to solid–liquid ratio and power for oil yield. The ellipse comprises the range of solid–liquid ratios greater than 1:30 at the lowest power values used (>50 W). Therefore, highlighted in red is the region optimized for *Moringa oleifera* oil yield. The optimal point determined from Equation (3) was found at a solid–liquid ratio of approximately 1:34 and a power of 175 W. The experimental data found in Table 3 are confirmed by the results presented in Figure 4 with the best results obtained in runs 6 and 4. Therefore, the response surface methodology was able to confirm the experimental data found in the previous experiment, which were able to predict the optimum region above the levels studied, and also made it possible to find the optimum region for oil yield.

### 3.3. Comparison of Moringa oleifera Oil 

#### 3.3.1. Quantification of Fatty Acids from *Moringa oleifera* Oil

The quantification of the fatty acids presents in *Moringa oleifera* oil, extracted by different methods and solvents, was conducted using gas chromatography with flame ionization detection (GC-FID). The results in Table 5 show the relative percentage composition of the fatty acids present in *Moringa oleifera* oil. The relative percentage of fatty acids was determined based on the retention time of the sample that of the corresponding methyl ester standards.

The gas chromatographic analysis revealed that the crude oil of *Moringa oleifera* is composed of a range of fatty acids, including myristic (C14:0), palmitic (C16:0), palmitoleic (C16:1), stearic (C18:0), oleic (C18:1), linoleic (C18:2), arachidonic (C20:0), gadoleic (C20:1), behenic (C22:0), and lignoceric (C24:0). As illustrated in Table 5, the quantitative analysis reveals that the oil of *Moringa oleifera* is predominantly composed of oleic acid (85% to 88%), followed by stearic acid (2% to 4%), palmitic acid (3% to 4%), and behenic acid (2% to 3%). Additionally, the presence of other unsaturated fatty acids is noteworthy, including palmitoleic (1% to 2%), linoleic, and gadoleic (both with less than 1%). Finally, the saturated fatty acids myristic and lignoceric were present in quantities of less than 0.55% of the relative percentage composition. The comparison proved that oils had similar profiles, reiterating the use of microwave with ethanol, as it is a timesaving and environmentally friendly technique.

#### 3.3.2. *Moringa oleifera* Oil Yield 

**Figure 5:**
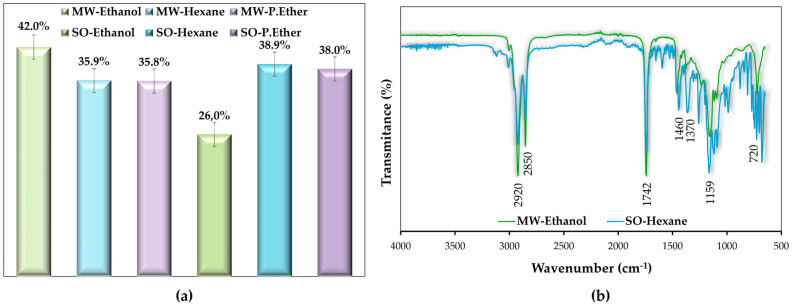
(**a**) *Moringa oleifera* oil yield (%) extracted by different methods. (**b**) Functional groups present in *Moringa oleifera* oil by FTIR. The combinations of extraction methods and solvents were microwave (MW) with ethanol (MW–Ethanol), hexane (MW–Hexane), and petroleum ether (MW–P.Ether); and Soxhlet (SO) with ethanol (SO–Ethanol), hexane (SO–Hexane) and petroleum ether (SO–P.Ether).

The extraction percentage of *Moringa oleifera* oil yield was evaluated using the microwave and Soxhlet methods, using ethanol, hexane, and petroleum ether as solvents, in Figure 5a. The results of the ethanol extraction show an increase in oil yield from 26% to 42% in the Soxhlet method compared to the microwave method. Conventional extraction by Soxhlet with hexane and petroleum ether showed oil yields of 38.9% and 38%, respectively; these data are consistent with the literature [6]. Microwave-assisted extraction with hexane and petroleum ether showed oil yields of 35.9% and 35.8%, respectively. 

These data show that the method and type of solvent can influence oil extraction, and this study highlights the potential of using microwaves as an alternative to speed up the process and ethanol as a greener solvent capable of conducting electromagnetic waves more efficiently by polarization than less polar solvents such as hexane and ethers. In this work, microwave-assisted extraction with ethanol had a higher optimized oil yield than the conventional method with hexane and petroleum ether. In addition, it has already been seen that the composition of the fatty acids present is similar in the different techniques. Thus, this protocol is an efficient and sustainable alternative for oil extraction, making it possible to extract non-polar molecules, such as oil, from the polar molecules of ethanol. The electromagnetic waves generated by microwaves allow for changes in mass transfer, resulting in greater efficiency when compared to traditional heating methods.

#### 3.3.3. Characterization of *Moringa oleifera* Oil by FTIR

FTIR analyzed the functional groups present in *Moringa oleifera* oil. The oil obtained by microwave-assisted extraction with ethanol (MW–Ethanol) was compared with the more conventional method, Soxhlet with hexane (SO–Hexane), as shown in Figure 5b.

The spectrum obtained in Figure 5 indicates the absence of peaks after 3000 cm^−1^, indicating low concentrations of impurities such as glycerol [24]. Additionally, the absence of a peak in the region of ∼3320 cm^−1^ indicates the absence of residual moisture in the samples, which confirms the moisture data obtained in this study [25]. The water content in the oil, determined by the Karl Fischer method, was 0.5%. These results agree with those found in the literature [24]. Furthermore, the absence of a rounded peak in the 3900–3200 cm^−1^ region suggests that the alcohol or phenol (OH-stretch) is not present [26]. Hence, it can be concluded from the absence of change at this wavelength that the alcohol used in the extraction has evaporated.

The peaks at 2920 cm^−1^ and 2850 cm^−1^ are attributed to the symmetrical and asymmetrical stretching of the C-H group of CH_2_ present in fatty acids [27]. The peak at 1742 cm^−1^ corresponds to the stretching of the C-O bonds of the ester functional groups of lipids and fatty acids. The characteristic absorption bands of amides may be linked to the repeating units of polypeptides and proteins. The peak observed at 1641 cm^−1^ are attributed to the amide I region, which represents the secondary structural component of proteins with a double-bond CO stretching movement that characterizes the protein conformation [25]. Between 1770 and 1660 cm^−1^, the observed peak confirms the presence of the ester functional group. Intense bands corresponding to the stretching of the C=O bond, a carbonyl group characteristic of proteins and fatty acid structures, were also detected [24,27].

The peak at 1460 cm^−1^ indicates the presence of an aromatic C=C bond [6]. The significant differences in the FTIR spectra of the samples in the 1460–652 cm^−1^ wavenumber band have been attributed to the reduction of polysaccharides, lignin, and phenolic and aromatic compounds that may have occurred during oil extraction [25]. Additionally, OH bending vibrations of phenols have been observed in the region with a peak at 1370 cm^−1^ [26]. The 1159 cm^−1^ peak is attributed to diacylglycerol esters and signifies stretching, while the 720 cm^−1^ peak is a result of asymmetric deformation of the CH_2_ group [28].

## 4. Discussion

New insights into microwave-assisted oil extraction removal from *Moringa oleifera* Lam. seeds using ethanol were highlighted in this work. Compared to the conventional method, microwave-assisted oil extraction shows the advantage of reducing the time and automating the process. The time reduction was observed in this experimental scale. In the investigation, the oil yield and fatty acid composition obtained from *Moringa oleifera* seeds extraction were similar. The ethanol, due to its polar nature, showed a lower oil yield in the conventional method. However, the use of microwave-assisted ethanol enabled high efficiency in removing nonpolar molecules from the oil. This highlights the preference for the use of ethanol over hexane and petroleum ether, which are toxic solvents [17]. 

The acceleration observed in irradiation process and the high oil yield may be attributed to the synergistic combination of transport phenomena. In microwave-assisted extraction, heat and mass transfer occur through diffusion, with both moving from the inside to the outside of the solid matrix. This contrasts with conventional heating, where heat is transferred by conduction from the heating medium directly to the molecule through contact with the heat source [29]. In microwave-assisted extraction, electromagnetic waves can penetrate materials and interact primarily with polar constituents, as observed with ethanol as a polar solvent. This is because the process dissipates energy through dipole polarization and ionic conduction. Dipole polarization breaks weak hydrogen bonds, resulting in a temperature increase. Ionic conduction enhances solvent penetration into the matrix and facilitates solvation of target compounds [30,31].

In a recent study, Nebolisa et al. (2023) [17] employed a novel approach to microwave-assisted oil extraction from *Moringa oleifera* Lam. seeds, using petroleum ether as a solvent [17]. This method was compared with conventional techniques. However, the comparison of green solvents with petroleum ether has not yet been evaluated. In this sense, the advantages of microwave-assisted ethanol extraction include the high solubility of oils and the lower cost of solvents. Furthermore, processes that use toxic solvents generate products and wastes that, when released into the environment, cause harmful effects to the environment and human and animal health [15]. 

Prat et al. (2016) [16] drew up a guide to selecting common solvents based on a benchmark of publicly available solvents. A total of 51 solvents were considered classic and classified into four categories: recommended, problematic, hazardous, and highly hazardous. This classification was developed to encourage the use of recommended solvents, such as ethanol, and inhibit the use of solvents considered dangerous, such as hexane, or highly hazardous, such as petroleum ether [16]. Ethanol is a polar compound that is safely available to the human body and can be used as a cheap solvent in oil extraction [15]. 

In statistical analysis, response surfaces are three-dimensional representations demonstrating how the response varies alterations in the independent variables. Contour curves are two-dimensional projections of these surfaces, wherein each line represents a constant value of the response, thereby facilitating the visualization of interactions between variables (Figure 2 and Figure 4) [32]. Therefore, response surface methodology (RSM) not only confirmed the predicted optimal conditions but also identified the optimal region for achieving the highest oil extraction yield. This approach demonstrated the effectiveness of microwave-assisted extraction in enhancing process energy efficiency compared to conventional methods, as illustrated in Figure 4 and the fundamentals of microwave extraction [27]. Furthermore, the CEM Corporation Model Discover SP microwave ensured automation and control of the power, temperature, and pressure conditions [33].

It is important to emphasize that in terms of productivity, microwave-assisted extraction presents a significant reduction in time, from 8 h to 1 h. In the microwave samples, although the oil yield, quantification of fatty acids, and identification of functional groups were similar to the Soxhlet samples, these data were obtained in one eighth of the time.

## 5. Conclusions

This research evaluated the extraction of *Moringa oleifera* oil from the effect of different variables of the microwave-assisted ethanol process and compared this optimized method with the conventional method (Soxhlet) and the usual toxic solvents. The results showed that, in microwave-assisted extraction with ethanol, the percentage of oil yield was mainly influenced by the solid–liquid ratio and the power, while temperature had negligible effects. Validation of the data obtained in the experimental design confirmed that the highest oil yield of 42% was in line with that obtained using conventional extraction methods under the best experimental conditions. The calculated optimum point showed experimentally that a lower ratio (1:34) and a lower power (175 W) could also be used to achieve optimum oil yield, as seen in the RSM. Therefore, the DCCR and RSM allowed not only cost reduction concerning the number of experiments but also savings in the amount of solvent and energy (power). Microwave-assisted ethanol proved to be a more viable alternative to hexane and petroleum ether and the Soxhlet extraction system, reducing the processing time from 8 h to 1 h, allowing the use of milder temperatures, and preserving the properties of *Moringa oleifera* oil, which is rich in oleic acid (85–88%). These data prove that electromagnetic waves directly affect the direction of mass transfer, improving the energy efficiency of the process and favoring the use of polar molecules in microwaves. It can be concluded that ethanol acted as an efficient green solvent, effectively releasing the nonpolar molecules present under microwave exposure. Therefore, microwave-assisted extraction of *Moringa oleifera* oil with ethanol allows for a more environmentally friendly extraction process without losing efficiency and productivity, resulting in a more sustainable product. 

## Figures and Tables

**Table 1:** Independent variables and five levels of CCRD 2^3^ and CCRD 2^2^.

	Independent Variables	Code	Levels				
			α (−1.68)	(−1)	0	(+1)	α (+1.68)
CCRD 2^3^	Solid–liquid ratio (*m*/*v*)	x_1_	1:4	1:8	1:14	1:20	1:24
	Power (W)	x_2_	50	100	175	250	300
	Temperature (°C)	x_3_	45	50	55	60	65
	Independent variables	Code	Levels				
			α (−1.41)	(−1)	0	(+1)	α (+1.41)
CCRD 2^2^	Solid–liquid ratio (*m*/*v*)	x_1_	1:10	1:14	1:24	1:34	1:38
	Power (W)	x_2_	50	100	175	250	300

**Table 2:** CCRD 2^3^ matrix for microwave-assisted oil extraction and its variables at the coded levels.

Run	Independent Variables			Dependent Variables
	Solid–Liquid Ratio (*m*/*v*) x_1_	Power (W) x_2_	Temperature (°C) x_3_	Oil Yield (%) Y
1	−1 (1:8)	−1 (100)	−1 (50)	10.40
2	1 (1:20)	−1 (100)	−1 (50)	27.90
3	−1 (1:8)	1 (250)	−1 (50)	14.10
4	1 (1:20)	1 (250)	−1 (50)	32.90
5	−1 (1:8)	−1 (100)	1 (60)	11.40
6	1 (1:20)	−1 (100)	1 (60)	31.40
7	−1 (1:8)	1 (250)	1 (60)	10.50
8	1 (1:20)	1 (250)	1 (60)	32.10
9	−1.68 (1:4)	0 (175)	0 (55)	9.50
10	1.68 (1:24)	0 (175)	0 (55)	31.60
11	0 (1:14)	−1.68 (50)	0 (55)	15.60
12	0 (1:14)	1.68 (300)	0 (55)	24.80
13	0 (1:14)	0 (175)	−1.68 (45)	24.10
14	0 (1:14)	0 (175)	1.68 (65)	24.90
15	0 (1:14)	0 (175)	0 (55)	23.80
16	0 (1:14)	0 (175)	0 (55)	29.00
17	0 (1:14)	0 (175)	0 (55)	26.40
18	0 (1:14)	0 (175)	0 (55)	25.10

**Table 3:** Matrix for microwave-assisted oil extraction and its variables in the coded levels of CCRD 2^2^, with 4 axial points and 3 central points.

Run	Independent Variables		Dependent Variables
	Solid–Liquid Ratio (*m*/*v*) x_1_	Power (W) x_2_	Oil Yield (%) Y
1	−1 (1:14)	−1 (100)	20.80
2	1 (1:34)	−1 (100)	39.20
3	−1 (1:14)	1 (250)	24.40
4	1 (1:34)	1 (250)	39.90
5	−1.41 (1:10)	0 (175)	14.20
6	1.41 (1:38)	0 (175)	42.00
7	0 (1:24)	−1.41 (50)	35.00
8	0 (1:24)	1.41 (300)	37.90
9	0 (1:24)	0 (175)	38.80
10	0 (1:24)	0 (175)	39.20
11	0 (1:24)	0 (175)	37.60

**Table 4:** ANOVA of the effects model on *Moringa oleifera* oil yield.

Variation Source	Sum of Squares	Degrees of Freedom	Mean Square	Fcalc	*p*-Value
Regression	853.3	5	170.7	107.5	0.00004
Residuals	7.9	5	1.6		
Lack of Fit	6.6	3	2.2	3.1	0.25026
Pure Error	1.4	2	0.7		
Total	861.3	10			

**Table 5:** Relative percentage composition of the fatty acids in *Moringa oleifera* oil as shown by gas chromatography for three solvents (ethanol, hexane, and petroleum ether). The combinations of extraction methods and solvents were microwave (MW) with ethanol (MW-Ethanol), hexane (MW-Hexane), and petroleum ether (MW-P.Ether); and Soxhlet (SO) with ethanol (SO-Ethanol), hexane (SO-Hexane), and petroleum ether (SO-P.Ether).

Fatty Acids	Determined Values (Method-Solvent)					
	MW–Ethanol	MW–Hexane	MW–P. Ether	SO–Ethanol	SO–Hexane	SO–P. Ether
C14:0	0.55 ± 0.17	0.06 ± 0.02	0.11 ± 0.01	0.13 ± 0.03	0.07 ± 0.02	0.24 ± 0.03
C16:0	3.47 ± 0.27	3.50 ± 0.17	3.55 ± 0.01	3.95 ± 0.04	3.45 ± 0.02	3.52 ± 0.17
C16:1	1.38 ± 0.16	1.19 ± 0.06	2.47 ± 0.2	1.54 ± 0.24	1.41 ± 0.14	1.65 ± 0.10
C18:0	2.78 ± 0.08	2.98 ± 0.02	2.20 ± 0.01	3.11 ± 0.59	3.03 ± 0.74	4.15 ± 0.03
C18:1	87.89 ± 0.18	87.78 ± 0.41	87.11 ± 1.81	87.49 ± 1.03	88.11 ± 1.38	85.62 ± 0.11
C18:2	0.32 ± 0.01	0.25 ± 0.08	0.41 ± 0.00	0.30 ± 0.01	0.28 ± 0.00	0.35 ± 0.09
C20:0	1.09 ± 0.12	1.17 ± 0.01	1.96 ± 0.04	1.19 ± 0.18	1.15 ± 0.30	1.61 ± 0.06
C20:1	0.32 ± 0.01	0.28 ± 0.02	0.52 ± 0.01	0.26 ± 0.02	0.27 ± 0.03	0.31 ± 0.01
C22:0	1.99 ± 0.33	2.51 ± 0.14	1.57 ± 0.01	2.02 ± 0.05	2.18 ± 0.17	2.53 ± 0.17
C24:0	0.24 ± 0.00	0.28 ± 0.05	0.11 ± 0.00	0.06 ± 0.01	0.05 ± 0.02	0.11 ± 0.01
ΣSaturated	10.14	9.50	9.50	10.47	9.93	12.18
ΣUnsaturated	89.54	89.25	90.10	89.30	89.79	87.59

## Data Availability

The original contributions presented in the study are included in the article, further inquiries can be directed to the corresponding author.
